# Pregnancy outcomes in women growing up with perinatally acquired HIV in the United Kingdom and Ireland

**DOI:** 10.7448/IAS.17.4.19693

**Published:** 2014-11-02

**Authors:** Laura Byrne, Claire Thorne, Caroline Foster, Pat Tookey

**Affiliations:** 1 Policy and Practice Programme, UCL Institute of Child Health, Population, London, UK; 2 Paediatric Infectious Diseases, Imperial College Healthcare NHS Trust, London, UK

## Abstract

**Introduction:**

In the United Kingdom and Ireland more than 40% of individuals living with perinatally acquired HIV are now aged >16. Globally, increasing numbers of women with perinatally acquired HIV are becoming pregnant, but data on fertility and pregnancy outcomes is scarce. We present pregnancy outcome data for this emerging cohort.

**Methods:**

Pregnancies in diagnosed HIV-infected women in the United Kingdom and Ireland, and children diagnosed with HIV, are reported to the National Study of HIV in Pregnancy and Childhood. We analyzed data on pregnancies in women diagnosed aged ≤13 with perinatally acquired HIV, reported by June 2014.

**Results:**

A total of 759 females born before 2001, diagnosed with perinatally acquired HIV aged ≤13 years, and in care in the UK and Ireland have been reported. Forty-four of these (6%) have had at least one pregnancy reported, with nineteen 2nd and four 3rd/4th pregnancies. Women's year of birth ranged from 1985 to 1996; 60% of women were UK/Irish-born and 39% African-born. Twenty one percent were diagnosed at <2 years, 39% at 2–7 and 41% at 8–13 years. Nine pregnancies were conceived in 2005–07, 22 in 2008–10 and 36 in 2011–13. Median age at conception of first pregnancy was 19 years. CD4 count was >500 cells/µL in 36% of first pregnancies, 350–499 in 15% and <350 in 49%. Women were on antiretroviral therapy (ART) at conception in 71% of pregnancies. There were 51 singleton live births, 2 miscarriages, 9 terminations and 5 continuing to term. In 17 live births to women not on ART at conception, median gestational age at start of ART was 17 weeks (range 3–29). HIV viral load was <50 copies/mL near delivery in 64% of live births, 51–1000 in 31% and >1000 in 5%. Forty four percent of live births were delivered by elective caesarean section (CS), 27% by emergency CS, 27% by planned vaginal delivery and with one unplanned vaginal delivery. Of 29 live births with viral load <50, 31% were delivered by elective CS, 17% by emergency CS and 52% by vaginal delivery. Fifteen percent of infants were delivered at 32–36 weeks gestation, and 2% at 30 weeks; 16% weighed 1.5–2.5 kg and 16% weighed <1.5 kg. Among 38 of the 51 infants where infection status is already reported, one is perinatally infected.

**Conclusions:**

Currently at least 6% of perinatally infected women in care in the UK and Ireland have experienced one or more pregnancies. Linking paediatric, pregnancy and second generation data will enable further monitoring of pregnancy outcomes in this newly emerging population.

